# Patellofemoral Pain Syndrome: Focused Vibrations Plus Kinesiotaping with Insights into Radiological Influences—An Observational Study

**DOI:** 10.3390/jfmk10010002

**Published:** 2024-12-24

**Authors:** Gabriele Santilli, Milvia Martino, Patrizia Pacini, Francesco Agostini, Andrea Bernetti, Luca Giuliani, Giovanni Del Gaudio, Massimiliano Mangone, Vincenzo Colonna, Mario Vetrano, Maria Chiara Vulpiani, Giulia Stella, Antonello Ciccarelli, Samanta Taurone, Antonio Franchitto, Carlo Ottonello, Vito Cantisani, Marco Paoloni, Pietro Fiore, Francesca Gimigliano

**Affiliations:** 1 Department of Movement, Human and Health Sciences, Division of Health Sciences, University of Rome “Foro Italico”, 00135 Rome, Italy; 2 Department of Radiological Sciences, Oncology and Pathology, Policlinico Umberto I, Sapienza University, 00185 Rome, Italy; 3 Department of Anatomical and Histological Sciences, Legal Medicine and Orthopedics, Sapienza University, 00185 Rome, Italy; 4 Department of Biological and Environmental Sciences and Technologies, University of Salento, 73100 Lecce, Italy; 5 Physical Medicine and Rehabilitation Unit, Sant’Andrea Hospital, Sapienza University of Rome, 00189 Rome, Italy; 6 Neurorehabilitation and Adapted Physical Activity Day Hospital, Bambino Gesù Children’s Hospital, IRCCS, 00165 Rome, Italy; 7 Fisiocard Medical Centre, Via Francesco Tovaglieri 17, 00155 Rome, Italy; 8 Neurorehabilitation Unit, Institute of Bari, Istituti Clinici Scientifici Maugeri IRCCS, 70124 Bari, Italy; 9 Department of Physical and Mental Health and Preventive Medicine, University of Campania “Luigi Vanvitelli”, 80100 Naples, Italy

**Keywords:** PFPS, patellofemoral pain, patellofemoral osteoarthritis, anterior knee pain, chondromalacia, musculoskeletal disorder, prevention, MRI, US, KOOS, VAS

## Abstract

Background: This observational study investigates the efficacy of combining local muscle vibration (LMV) therapy and kinesiotaping using the McConnell method (KMcCM) in patients with patellofemoral pain syndrome (PFPS). PFPS is a prevalent knee condition characterized by anterior or medial knee pain exacerbated by activities that overload the patellofemoral joint. Objective: The primary aim of this study was to evaluate the effectiveness of LMV combined with KMcCM in reducing pain and improving function in PFPS patients. Methods: A total of 52 participants, aged 25–85, with PFPS were included. Participants underwent LMV and KMcCM treatments three times weekly for three weeks. Pain and function were assessed using the Visual Analog Scale (VAS) and the Knee Injury and Osteoarthritis Outcome Score (KOOS) at baseline (T0) and six months post-treatment (T1). Radiological assessments of patellar alignment and biomechanics were also conducted through dynamic MRI. Results: Significant pain reduction and functional improvements were observed across all age groups. Notably, younger participants showed greater improvement compared to older participants. Among women, those in the younger age group experienced more substantial reductions in VAS scores compared to their older counterparts. KOOS scores improved significantly, indicating enhanced knee function overall. A significant decrease in VAS scores from T0 to T1 was observed across all patellar alignment groups, signifying a reduction in pain levels. However, Group 2 (Laxation and Subluxation) experienced the most substantial reduction in VAS scores at T1 compared to the other groups. These results suggest that the combination of LMV and KMcCM may be particularly effective in addressing biomechanical abnormalities associated with patellar maltracking and enhancing VMO muscle contraction, leading to more substantial improvements in these patients. Conclusions: The combination of LMV and KMcCM demonstrates promising efficacy in reducing pain and improving knee function in PFPS patients, with age and gender influencing treatment outcomes. The most significant improvements were observed in younger individuals and those with specific patellar alignment issues, highlighting the potential of this combined approach for the targeted treatment of PFPS.

## 1. Introduction

Patellofemoral pain syndrome (PFPS) is a pathological condition of the knee, commonly found in young adults, and is characterized by diffuse pain in the anterior and/or medial part of the knee that occurs during weight-bearing activities and knee flexion [1]. A recent consensus established that the primary criterion for diagnosing PFPS is peri- or retro-patellar pain that worsens with activities that overload the patellofemoral joint when the knee is flexed, such as squatting, climbing or descending stairs, jogging, running, or jumping [2]. Additional but non-essential criteria include joint crepitus during flexed knee activities, tenderness on palpation of the patellar facets, mild effusion, pain with prolonged sitting, and discomfort when transitioning from sitting to standing [1]. It is estimated that approximately 11–17% of young patients presenting to a specialist with knee pain suffer from PFPS, and 25–40% of athletes are affected [2]. This syndrome is more common among athletes, with 25–40% of this population being affected [2]. PFPS can severely affect patients’ quality of life by limiting their ability to perform daily activities such as walking, climbing stairs, and maintaining an active lifestyle. Anxiety, depression, catastrophizing, and fear of movement may be elevated in individuals with PFPS and correlate with pain and reduced physical function [3]. However, PFPS is not limited to the athletic population or young adults; it is also a significant cause of knee pain among older adults, often resulting from age-related degenerative changes, reduced muscle strength, and altered biomechanics [4]. PFPS may also present with structural findings on imaging; particularly in older adults with osteoarthritis (OA) [5,6,7]. Regarding gender, females are twice as likely to develop PFPS compared to males [8]. PFPS has a multifactorial etiology [9]. Contributing factors can be classified into local joint issues, such as altered patellar tracking with patellar hypermobility [10], morphological alteration of the femoral trochlea and patella [11], delayed activation of the vastus medialis (VM) compared to the vastus lateralis (VL), and hypotrophy of the vastus medialis oblique (VMO) [12], as well as tightness in the iliotibial band, quadriceps femoris, hamstrings, and gastrocnemius muscles [13]. General factors include hip abductor and external rotator muscle weakness and altered foot positioning [14]. These factors collectively contribute to abnormal lower limb biomechanics [15]. Some studies have also shown that patients with PFPS are more likely to suffer from psychological conditions such as anxiety, depression, kinesiophobia, and dependency compared to control groups [16] due to errors or overloads during training [17]. Diagnosis of PFPS is primarily clinical and based on a detailed history and thorough physical examination [16]. Imaging is typically not necessary for diagnosis, but a 2016 meta-analysis revealed that magnetic resonance imaging (MRI) can identify some typical PFPS features, such as an increased bisect-offset during load-bearing with the knee at 0°, patellar tilt, and contact in the patellofemoral area [18]; ultrasound examination demonstrated high-value results in the diagnosis of PFS [19,20]. A real-time magnetic resonance imaging (MRI) study reported that the patella exhibits a relative lateral shift as the femur rotates into adduction and internal rotation during a squat or step-down maneuver in women with PFP [21]; the application of dynamic MRI allowed for an evaluation of the malalignment of the patella [22].

In 2018, the International Patellofemoral Research Network (iPFRN) published a consensus document on the management of patellofemoral pain (PFP), identifying a hip- and knee-focused exercise regimen as the approach with the highest level of evidence for effectiveness. Additionally, supplementary treatments such as patellar taping, manual therapy, and foot orthoses are recommended, though not as standalone treatments. Taping is a more recent technique, with the two most commonly used methods being McConnell taping and kinesiotaping [23,24]. Although their mechanisms differ (McConnell taping addresses the imbalance between VMO and VL, while kinesiotaping stabilizes the patella by medializing it), both have proven effective in reducing pain when combined with therapeutic exercise [25]. Furthermore, Lan et al. [26] combined the McConnell taping technique with kinesiotaping to correct patellar alignment. There is limited evidence in the literature regarding the use and effectiveness of instrumental physical therapies, such as ultrasound, laser, NMES, TENS, and biofeedback [27]. Similarly, there is little research on the effectiveness of orthoses in the treatment of PFPS compared to other treatments. The most commonly used orthoses are knee braces, which alter patellar positioning, or custom-made insoles to correct foot abnormalities [28].

Whole body vibration (WBV) training was shown to improve muscle strength, power, balance, flexibility, proprioception, and gait in both healthy adults and the elderly [29,30,31,32]. Previous studies have demonstrated that WBV training can alleviate knee pain, enhance knee function, and improve muscular performance in individuals with knee osteoarthritis (KOA) [33,34,35,36]. WBV can be an effective and alternative option as a training technique for PFP [37].

However, a decrease in vibration energy before attaining the target muscle, the high cost of associated equipment, and the limited portability of the WBV platform, are reasons to limit its application in clinical settings [38,39]. The local muscle vibration (LMV) is a method of neuromuscular training that could be directly applied to the targeted muscle belly or muscle–tendon via a lightweight handheld applicator and might be considered as a cost-effective alternative for WBV, and a review revealed the promising effect of LMV on pain, stiffness, function, and knee range of motion (ROM) improvements for individuals with knee osteoarthritis (OA) [40]. Therefore, LMV could be an effective and alternative option as a training technique for PFP.

Despite the proven benefits of hip- and knee-focused exercise regimens, many patients with PFPS experience incomplete pain relief and functional recovery [41], highlighting the need for adjunctive treatments. While whole-body vibration (WBV) shows promise, its high cost, limited portability, and reduced energy transfer restrict its use [39,40], underscoring the potential of local muscle vibration (LMV) as a cost-effective alternative. Combining LMV with kinesiotaping, particularly using the McConnell technique, may enhance patellar stabilization and prolong therapeutic effects, potentially improving outcomes. This study explores the synergistic potential of LMV and kinesiotaping.

This study is an observational study aiming to evaluate the effectiveness of combined local muscle vibration (LMV) therapy with kinesiotaping applied using the McConnell method and to assess their long-term impact on pain and overall lower limb function in patients with PFPS.

## 2. Materials and Methods

### 2.1. Study Design and Population

This observational study follows the ethics of the Helsinki Declaration, approved by La Sapienza University’s Institutional Review Board (Prot. 0032/2024—Approval Date: 10 January 2024). The STROBE checklist for observational studies was followed for this study. Informed consent forms were signed by all patients, and the data have been anonymized. All participants provided signed informed consent before the study, which included a specific section regarding the processing of their personal data for research purposes, ensuring anonymization to safeguard their privacy. The data of patients who underwent therapy were analyzed from a pre-existing dataset. PFPS was diagnosed based on clinical symptoms, physical examinations, and imaging studies [5]. All the patients who fulfilled the following selection criteria were considered eligible: (1) between 25 and 85 years old, this broad age range was selected to provide a translational perspective on the condition, which is observed across various age groups, as previously reported in other studies [19,42,43]; (2) diagnosis of PFPS based on clinical criteria of peri- or retro-patellar pain on at least 2 of the following activities such as prolonged sitting, squatting, ascending or descending stairs, kneeling, hopping, or running and a positive clinical patellar test [44,45,46] (Clarke’s test or patellar femoral grinding test); (3) had an MRI of the affected knee; and (4) both genders. Patients were excluded if they have the following: (1) neurological or rheumatological pathologies, (2) trauma to the lower limbs, (3) are taking pain-relieving drugs, (4) have neoplasm, or (5) recent orthopedic surgery. All eligible patients completed a demographic and clinical questionnaire that assessed age and gender, and the scales administered at T0 and at T1 after 6 months of therapy were: Visual Analog Scale (VAS) [47] and Knee Injury and Osteoarthritis Outcome Score (KOOS) [48]; the six-month follow-up was chosen to assess both immediate and long-term effects of LMV and KMcCM, capturing delayed improvements and ensuring the stability of the outcomes. Shorter follow-up periods may not fully reflect these effects. This time frame is also commonly used in musculoskeletal studies, ensuring comparability with the existing literature [2,49]. All therapies were carried out by the same therapist. Participants were instructed not to undergo any additional therapeutic interventions, including physical therapy or pharmacological treatments with nonsteroidal anti-inflammatory drugs (NSAIDs), during the follow-up period after the intervention. Compliance with these instructions was emphasized during the initial recruitment and reinforced during telephone follow-up.

### 2.2. Intervention

**Figure 1:**
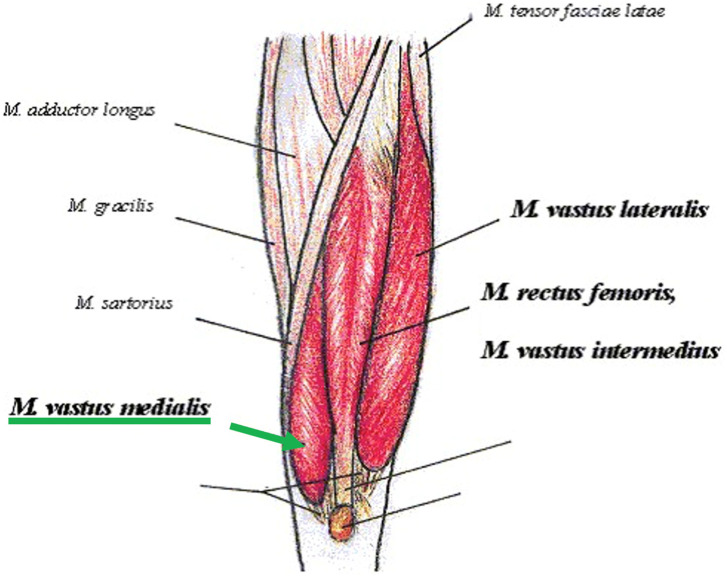
Schematic representations of muscle of thigh with vastus medialis (arrow green). Image by Alfred Grudszus, licensed under CC BY-SA 3.0 and GNU Free Documentation License (GFDL).

**Figure 2:**
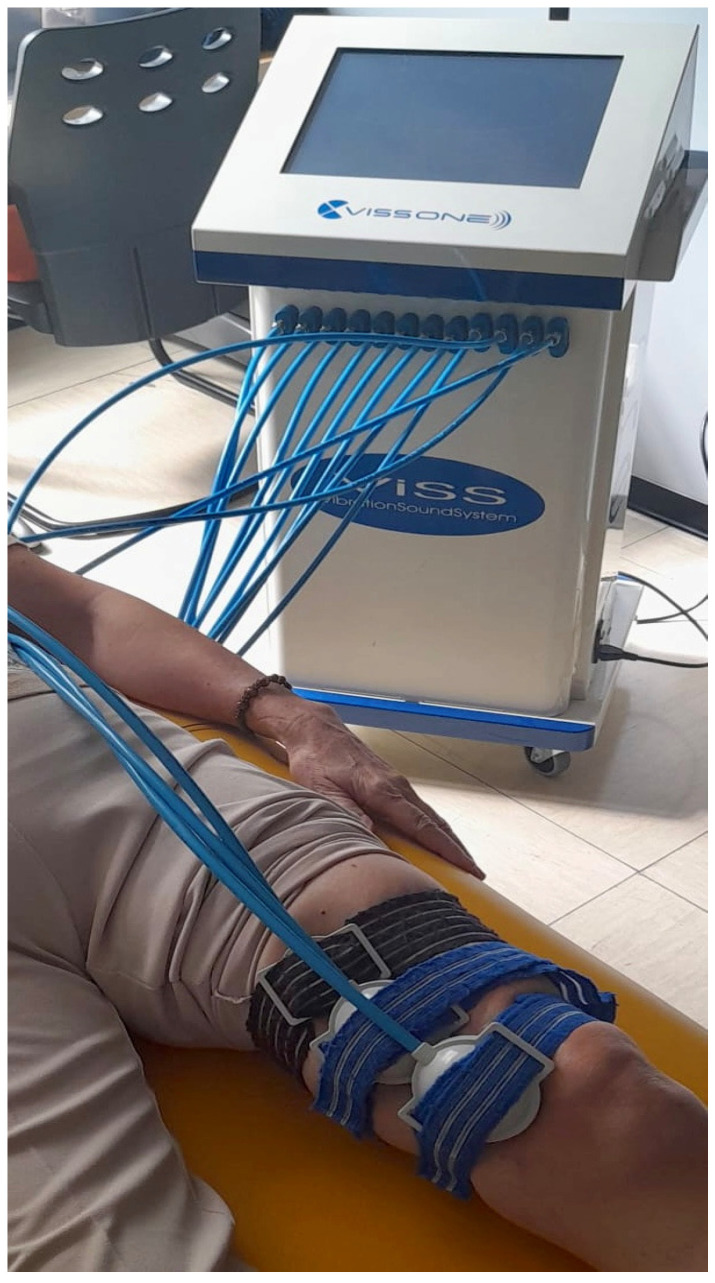
LMV Probe Application Protocol.

The LMV protocol consisted of the application of local high-intensity vibrations on the homolateral thigh of the PFP knee using the VISS apparatus. The VISS device (Vissman, Rome, Italy) is a tool capable of producing acoustic waves of different frequencies without affecting the set width. The device is not an acoustic wave generator, but rather a flux modulator, and has two components. These is a compressor delivering pressure in the range 0–400 millibar and a modulator producing an oscillatory air flux to create acoustic waves through a two-way rotating valve. The transducer develops a time-modulated sinusoidal wave (300 Hz) [50]. The VISS device is commonly used in musculoskeletal pathology, which, at a frequency of 300 Hz, represents an adequate stimulus for muscle–tendon proprioceptors, muscle spindles, and Golgi tendon organs, can significantly improve muscle strength, decrease muscle tone, disability, and pain [51,52]. During the 30 min of the application of vibrations, subjects were invited to avoid isometric contractions of the treated muscle. The protocol required that three probes producing local mechanoacoustic vibratory stimulation were applied on the skin of the distal part of the quadriceps and on the muscle belly of the Vastus medialis (Figure 1 and Figure 2). The focal vibrations were applied three times a week for three weeks.

**Figure 3:**
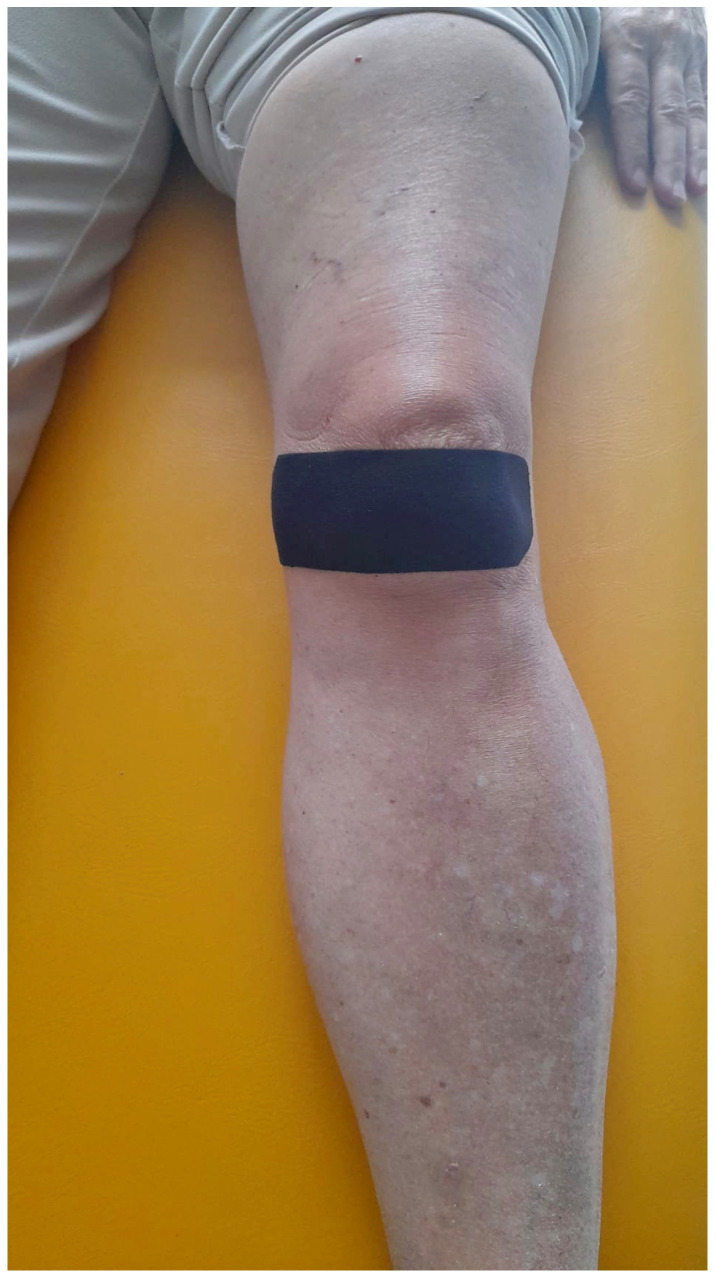
Kinesiotaping application protocol according to McConnell method.

After the LMV protocol, kinesiotaping was applied using the McConnell method (KMcCM) [53], where the patella was manually placed medially and maintained in that position with Tex Tape (Kinesio Holding Corp, Albuquerque, NM, USA) (Figure 3). Specifically, as noted in a recent review, the kinesiotaping technique used for muscles can relieve pain but cannot change patellar alignment, unlike McConnell taping. Both patellar taping techniques are used differently for PFPS patients and substantially improve muscle activity, motor function, and quality of life [25]. All procedures were performed by the same therapist who was blinded to the patients’ data. The therapist who administered the treatments underwent specific training to ensure consistency in both the intensity of the LMV therapy and the kinesiotaping technique. Both interventions were applied strictly according to a pre-defined protocol, which detailed the parameters for LMV settings, taping placement, and the duration of application. To ensure fidelity to the protocol, each session was documented in a clinical diary, recording key details such as LMV intensity and the taping method. The KMcCM was applied three times a week for three weeks. The VAS and KOOS were administered before treatments and at T1 after six months from treatments.

#### Outcomes

The outcome data were collected by a physiatrist. The Visual Analog Scale (VAS) comprises a 100 mm horizontal line, with “no pain” denoted at the left end (score: 0) and “pain as severe as possible” at the right end (score: 10). Patients were instructed to place a hatch mark on the line corresponding to their current pain level, both at rest and during their most painful movement. The VAS score was subsequently determined by measuring the distance in millimeters between the left endpoint and the patient’s mark [47]. The Knee Injury and Osteoarthritis Outcome Score (KOOS) is a patient-reported outcome measure designed to evaluate symptoms and functional limitations related to knee injuries and osteoarthritis. KOOS includes five subscales: pain, other symptoms (such as swelling and mechanical issues), activities of daily living (ADL), sports/recreation, and quality of life. Each subscale is scored on a 0–100 scale, with higher scores indicating better outcomes. KOOS is particularly useful in monitoring changes over time in response to treatments like surgery or physical therapy and is widely used in both clinical and research settings to assess knee function in various populations [22]. The decision to administer VAS and KOOS was based on previous studies in PFPS that have confirmed their applicability [54,55,56,57]. The application of dynamic MRI allowed for an evaluation of the malalignment of the patella [22]; patellar alignment was categorized into three groups based on imaging results: “0” normal alignment, “1” lateral tilt or lateral hyperpressure, and “2” lateral subluxation or dislocation [58,59,60]. Each subject underwent a dynamic MRI with the knee positioned in varying degrees of flexion to assess patellar movement during load-bearing conditions. This allowed for a comprehensive assessment of the patellofemoral joint’s biomechanics, which is critical for diagnosing different degrees of malalignment [61]. Furthermore, were assessed the articular cartilage thickness at the medial and lateral femoral condyles [62], the medial patellofemoral ligament thickness [63], and the presence of alterations in the medial and lateral menisci; a binary outcome was assigned to both the lateral and medial meniscus, indicating the presence or absence of meniscal pathology, specifically, the meniscus was classified as intact (normal, without degenerative changes, or not torn but with degenerative findings) or torn (with radial, longitudinal, or fracture lines visible in at least three slices, or with morphological deformities) [64]. The MRI findings were independently reviewed by two experienced radiologists to ensure consistent classification according to these pre-defined categories.

### 2.3. Statistical Analysis

Power analysis was performed using GPower (v.3.1.9.2, Franz Faul, Germany). Based on previous results by Agostini et al. [65], we calculated the sample size for a two-tailed test with an α level of 0.05 and a 95% confidence interval (CI). For a desired power of 95% (β = 0.05), the minimum required sample size was 24 participants. The acceptable precision level was determined with a standard deviation (SD) of 1.5 points. A 95% confidence level (α = 0.05) was specified, and an effect size of 0.93 was considered to determine the magnitude of practically significant differences. All statistical analyses were performed using SPSS version 28 (IBM Corp, Armonk, NY, USA). The normality of the data was confirmed using the Shapiro–Wilk test, enabling the application of parametric tests. Continuous variables are reported as means ± standard deviation (SD), and categorical variables are presented as frequencies and percentages. The General Linear Model (GLM) was employed to examine differences in outcomes over time (T0 to T1). Specifically, a series of two-way mixed ANOVAs were conducted to analyze the interaction between time and the following between-subject factors: (1) age groups: a two-way mixed ANOVA was performed with a within-subject factor of time (VAS T0 vs. VAS T1) and a between-subjects factor of age groups (<60 years vs. ≥60 years) [66,67,68,69]; (2) KOOS: a two-way mixed ANOVA was performed with a within-subject factor of time (VAS T0 vs. VAS T1) and a between-subjects factor of the delta KOOS, where participants were categorized into two groups based on the percentage increase in KOOS score from T0 to T1. Group 1 consisted of participants with less than a 60% increase in KOOS (non-clinical success), and Group 2 consisted of those with more than a 60% increase (clinical success). (3) Patellar Dynamic Alignment: A two-way mixed ANOVA was conducted with time (VAS T0 vs. VAS T1) as the within-subject factor and “Patellar Dynamic Alignment” as the between-subjects factor with these threshold values (0 = Neutral, 1 = Lateral Hyperpressure/Lateral Tilt, 2 = Laxation/Subluxation) [58,59,60]; finally, the difference in pain improvement between males and females, aged above or below 60 years, was analyzed, also considering a separate sample size to ensure statistical reliability. In particular, G*Power (v.3.1.9.2), developed by Franz Faul and colleagues at the University of Kiel, Germany, was used to perform a power analysis. Based on the data from Glass N et al. [70], a statistical power of 95% was assumed to detect a clinically significant difference of 2 points in the VAS-pain score between groups, using a one-tailed *t*-test. The standard deviation (SD) within each group was set at 0.5 points, and a confidence level of 95% (α = 0.05) was specified. The effect size (Cohen’s d) was calculated as 2, based on the ratio between the expected difference in means and the pooled standard deviation. Under these assumptions, a total of 14 participants (7 per group) were determined to be sufficient to detect the expected difference. Furthermore, the difference in pain improvement between different age groups, aged above or below 60 years, was analyzed, also considering a separate sample size to ensure statistical reliability To ensure the reliability of the analyses with subgroups, another sample size was calculated. In particular, G*Power (v.3.1.9.2, developed by Franz Faul and colleagues at the University of Kiel, Germany) was used to perform a power analysis. Based on the data from Buntin-Mushock et al. [71], a statistical power of 95% was assumed to detect a clinically significant difference of 2 points in the VAS-pain score between groups, using a one-tailed *t*-test. The standard deviation (SD) within each group was set at 0.5 points, and a confidence level of 95% (α = 0.05) was specified. The effect size (Cohen’s d) was calculated as 2, based on the ratio between the expected difference in means and the pooled standard deviation. Under these assumptions, a total of 14 participants (7 per group) were determined to be sufficient to detect the expected difference. In cases where a significant ANOVA result was found (*p* < 0.05), we performed post hoc analysis to determine which specific group differences contributed to the significance. Specifically, we used the Bonferroni correction for the post hoc test to adjust for multiple comparisons and minimize the risk of type I errors. To explore differences across the age subgroups, cross-tabulations were conducted to assess the distribution of clinical success within each subgroup. This analysis was followed by a two-way mixed ANOVA to determine if there were significant interactions between the subgroups and clinical success. To further investigate potential factors that could have influenced clinical success, we assessed the thickness of medial and lateral cartilage and MFPL thickness in the different subgroups using independent samples *t*-tests. These analyses aimed to determine if these elements contributed to the observed outcomes. Outliers and deviations within each subgroup were also evaluated to ensure the robustness of the results, with any extreme values being carefully considered and addressed.

## 3. Results

### 3.1. Patient Demographic and Clinical Characteristics

All patients had symptoms for at least three months. The study included participants with an average age of 66 ± 12.2 years. The average VAS T0 score was 6 ± 1.4, which decreased to 2.8 ± 1.9 at VAS T1. Similarly, the baseline KOOS T0 was 45.6% ± 9.6%, improving to 72.7% ± 15.3% at KOOS T1. The sample comprised 14 males and 38 females. Regarding patellar alignment, 15 participants (28.8%) exhibited normal alignment (coded as 0), 24 participants (46.2%) presented with lateral tilt or lateral hyperpressure (coded as 1), and 13 participants (25%) had lateral subluxation or dislocation (coded as 2). The measurement of the medial patellofemoral ligament thickness was 2 mm ± 0.7. The medial cartilage thickness was 1.8 mm ± 0.4, and the lateral cartilage thickness was 2 mm ± 0.5. Regarding the binary classification of the menisci, 35 medial menisci were classified as healthy and 17 as injured, while 41 lateral menisci were classified as healthy and 11 as injured. Concerning the age groups, 13 participants (25%) were under 60 years old, while 39 (75%) were 60 years or older. In terms of the delta KOOS threshold score, 11 participants had a delta KOOS treshold score below 59%, while 41 had a score above 60%. These results are resumed in Table 1.

We conducted a two-way mixed ANOVA with a within-subject factor of time (VAS T0-VAS T1) and a between-subjects factor of age group (1 = aged < 60 years and 2 = ≥ 60 years). There was a significant decrease in VAS scores from T0 to T1 for both groups. Specifically, in Group 1, the VAS score decreased from 5.6 ± 1.3 to 1.8 ± 1.3 (*p* < 0.001), while in Group 2, the VAS score dropped from 6 ± 1.4 to 3.2 ± 2 (*p* < 0.01), indicating a better improvement in the younger group. A significant interaction effect between age group and time on VAS measurements was observed, F(1, 50) = 3.71, *p* < 0.05, partial η^2^ = 0.07. At T1, the VAS score was significantly higher in Group 2 (3.2 ± 2) compared to Group 1 (1.8 ± 1.3), *p* < 0.05. These results are summarized in Table 2. The results of the contingency table analysis revealed that 61.5% of participants under 60 years achieved clinical success, compared to only 31% of those aged 60 years and older. This difference was statistically significant (*p* < 0.05). The Phi coefficient (−0.274) and Cramer’s V (0.274) further indicated a moderate negative association between age and clinical success. Upon examining potential contributing factors, we found that lateral trochlear cartilage thickness was significantly lower in participants over 60 years (1.95 ± 0.5 vs. 1.7 ± 0.4), while medial cartilage and MFPL thickness showed non-significant changes, with the medial cartilage slightly reduced and the MFPL thickened. These findings suggest that the cartilage thickness in the lateral trochlea may be an important factor to consider when interpreting the clinical outcomes.

We also conducted a two-way mixed ANOVA with a within-subject factor of time (VAS T0-VAS T1) and a between-subjects factor of delta KOOS, where participants were categorized into two groups based on the percentage increase in KOOS score from T0 to T1. Group 1 consisted of participants with less than a 60% increase in KOOS (non-clinical success), and Group 2 consisted of those with more than a 60% increase (clinical success) [72,73,74]. There was a significant decrease in VAS scores from T0 to T1 for both groups. Specifically, in Group 1, the VAS score decreased from 6.6 ± 1.1 to 2.7 ± 1.2 (*p* < 0.001), while in Group 2, the VAS score dropped from 5.7 ± 1.4 to 2.9 ± 2 (*p* < 0.01). A significant interaction effect between delta KOOS score and time on VAS measurements was observed, F(1, 50) = 4.3, *p* < 0.05, partial η^2^ = 0.08. At T1, there were no significant differences in VAS scores between Group 1 (2.7 ± 1.2) and Group 2 (2.9 ± 2). These results are summarized in Table 3.

A two-way mixed ANOVA was conducted with a within-subject factor of time (VAS T0-VAS T1) and a between-subjects factor of Patellar Dynamic Alignment (Group 0 = Neutral, Group 1 = Hyperpressure, Group 2 = Subluxation/Dislocation). There was a significant decrease in VAS scores from T0 to T1 for all groups. Specifically, in Group 0, the VAS score decreased from 5.3 ± 1.1 to 3.1 ± 2 (*p* = 0.01), in Group 1 from 6.1 ± 1.4 to 2.8 ± 2 (*p* < 0.001), and in Group 2 from 6.2 ± 1.5 to 2.5 ± 1.6 (*p* = 0.02). A significant interaction effect between Patellar Dynamic Alignment and time on VAS measurements was observed, F(2, 49) = 3.83, *p* = 0.028, partial η^2^ = 0.135. Post hoc analysis showed the greatest reduction in VAS scores at T1 occurred in Group 2 compared to Groups 1 and 0 (−3.7 vs. −3.2 vs. −2.2), *p* < 0.05. These results are summarized in Table 4.

Lastly, to assess gender differences, we conducted a two-way mixed ANOVA on the female subgroup with a within-subject factor of time (VAS T0-VAS T1) and a between-subject factor of age group (1 = Age < 60 years, 2 = Age ≥ 60 years). There was a significant decrease in VAS scores from T0 to T1 for both groups. Specifically, in Group 1 (younger), the VAS score decreased from 5.7 ± 1.6 to 1.3 ± 1.2 (*p* < 0.001), while in Group 2 (older), the VAS score dropped from 6.2 ± 1.5 to 3.2 ± 2.1 (*p* < 0.01). A significant interaction effect between age group and time on VAS measurements was observed, F(1, 36) = 4.9, *p* = 0.03, partial η^2^ = 0.12. At T1, the VAS score was significantly higher in Group 2 (3.2 ± 2.1) compared to Group 1 (1.3 ± 1.2), *p* < 0.05. These results are summarized in Table 5.

In contrast, the same analysis was conducted for the male subgroup, where no significant interaction between age group and time on VAS measurements was observed (F(1, 12) = 1.2, *p* = 0.3), indicating no significant differences in VAS reduction between younger and older males.

Correlation analysis revealed significant interactions between various components involved in osteoarthritis, particularly the lateral meniscus. A strong positive correlation was observed between the lateral meniscus binary values and the medial meniscus binary values (r = 0.6, *p* < 0.05), suggesting that the pathological involvement of one meniscus increases the likelihood of pathology in the other. Additionally, lateral meniscus binary values positively correlated with MPFL thickening (r = 0.4, *p* < 0.05) and patellar malalignment (r = 0.41, *p* < 0.05), while showing inverse correlations with the medial trochlear cartilage thickness (r = −0.41, *p* < 0.05) and the lateral trochlear cartilage thickness (r = −0.43, *p* < 0.05), indicating progressive cartilage thinning with increasing lateral meniscus pathology. Age also correlated positively with lateral meniscus involvement (r = 0.3, *p* < 0.05). No significant correlations were found between the medial meniscus and clinical success probability or gender.

Further analysis revealed that medial meniscus binary values correlated positively with MPFL thickening (r = 0.5, *p* < 0.05) and patellar malalignment (r = 0.4, *p* < 0.05), while inversely correlating with the medial trochlear cartilage thickness (r = −0.6, *p* < 0.05) and lateral trochlear cartilage thickness (r = −0.5, *p* < 0.05), as is consistent with cartilage degeneration. A positive correlation with female gender (r = 0.5, *p* < 0.05) was also observed. However, no significant associations were detected between the medial meniscus and clinical success probability or age.

### 3.2. Safety

Throughout the study period, no significant adverse effects related to the therapies were observed. Adverse events were monitored through patient self-reports and clinical evaluations were conducted at follow-up visits.

## 4. Discussion

The results of this study suggest that the combination of local muscle vibration (LMV) and kinesiotaping applied using the McConnell method (KMcCM) may be effective in reducing pain and improving lower limb function in patients with patellofemoral pain syndrome (PFPS). The decrease in VAS scores and the improvement in KOOS scores over time reflect significant positive outcomes for both younger and older participants, indicating that the interventions are beneficial across different age groups.

However, a closer examination reveals gender and age differences in response to treatment. For the female subgroup, there was a significant interaction between age and time, with younger women (<60 years) experiencing a greater reduction in VAS scores compared to older women (≥60 years). This interaction effect may be related to age-associated differences in tissue plasticity, recovery rates, or neuromuscular control, which are likely more pronounced in females than males, as suggested by previous studies [75,76]. These differences could partly stem from estrogen’s protective effects on connective and muscular tissue, particularly in younger women, which tend to diminish after menopause [77,78].

Interestingly, the same effect was not observed in the male subgroup, where no significant interaction between age and time was found. This disparity suggests that age-related physiological changes, such as decreased hormonal influence, may impact recovery trajectories differently in men and women. Estrogen, for instance, was shown to enhance pain modulation and promote faster recovery from musculoskeletal injuries in women, a mechanism that is not paralleled by testosterone in men [79].

Additionally, gender differences in pain perception and response to treatment can be influenced by various biopsychosocial factors. These include biological mechanisms such as hormonal fluctuations and their effects on nociception and inflammation; psychological factors such as coping strategies, emotional responses, and pain catastrophizing; and social influences such as gender roles and societal expectations regarding pain expression [80]. For example, younger women may perceive less pain due to higher endogenous estrogen levels, which can activate descending inhibitory pain pathways, whereas these pathways may not be as effectively engaged in older women or men [81].

Moreover, the lack of significant findings in the male subgroup raises the possibility of other confounders, such as baseline physical activity levels, differences in muscle fiber composition, or variations in adherence to the rehabilitation program. These factors, coupled with potential differences in reporting pain intensity between genders, highlight the need for further research to disentangle the complex interplay between biological, psychological, and social determinants of pain and recovery [82,83].

This suggests that age-related changes in pain perception or response to the intervention may vary by gender, potentially due to biological, hormonal, or psychosocial factors that influence pain and recovery mechanisms differently in men and women.

Given the advanced age of the subjects included in this study, it is essential to focus on certain structures that are indicative of osteoarthritis (OA) and play a significant role in patellofemoral pain syndrome (PFPS). These include the thickness of the medial and lateral trochlear cartilage, the condition of the menisci, and the thickness of the medial patellofemoral ligament (MPFL). Our findings showed a thickened MPFL compared to the normal range [84], aligning with previous studies that identified this ligament as a primary pain generator [85]. Additionally, both medial and lateral trochlear cartilage appeared thinner than the normal range [86,87,88]. This thinning, together with our observed correlations—such as the inverse relationship between meniscal pathology and trochlear cartilage thickness (r = −0.41 for medial, r = −0.43 for lateral, both *p* < 0.05)—highlights the need to consider PFP within the broader context of knee OA. These structural changes likely contribute to pain generation in PFPS, emphasizing their role as cofactors in the pathology.

These findings raise the possibility that the combined therapy may benefit not only patellofemoral pain syndrome (PFPS) but also symptoms associated with osteoarthritis (OA), particularly in older patients where structural changes such as cartilage thinning and meniscal pathology are more common. However, given the overlap in structural and symptomatic features between PFPS and OA, further studies are needed to clarify the extent to which the observed improvements are attributable to addressing PFPS alone versus a broader impact on OA-related changes. Future research should aim to differentiate the specific effects of local muscle vibration (LMV) and kinesiotaping (KMcCM) on PFPS and OA by including distinct patient populations with isolated PFPS, isolated OA, or comorbid conditions. This could provide a clearer understanding of the mechanisms underlying the therapy’s effects and guide its application in clinical practice.

Additionally, the improvement in KOOS scores indicates that the combined therapy not only reduces pain but also enhances daily function, including activities of daily living and quality of life, as suggested by other papers that have correlated pain scales with functionality scale improvements in pain [65,89]. This is particularly important given the impact of PFPS on mobility and overall function in affected individuals. The results show that both groups experienced a significant reduction in pain (VAS) from T0 to T1, even though the group with more than a 60% improvement in KOOS had greater overall improvement. However, there was no significant difference in pain levels between the two groups at T1. This suggests that, in daily clinical practice, both groups benefited similarly from the treatment, with pain reduction occurring regardless of the level of improvement in functional scores.

The application of LMV and KMcCM presents practical advantages and challenges that warrant consideration. Unlike whole-body vibration (WBV), which requires bulky, expensive, and less portable equipment, LMV is cost-effective and can be easily applied using a lightweight, handheld device directly targeting the muscle or tendon [38,39]. This feature enhances its feasibility in various clinical and outpatient settings. However, scalability may depend on ensuring proper therapist training to guarantee consistent and effective application.

The two-way mixed ANOVA demonstrated a significant decrease in VAS scores from T0 to T1 for all patellar alignment groups, indicating that pain levels improved across the board. The interaction effect between Patellar Dynamic Alignment and time was significant, suggesting that the rate of pain reduction varied depending on the group. Post hoc analysis revealed that Group 2 (Laxation and Subluxation) experienced the most substantial reduction in VAS scores at T1 compared to the other groups. This finding suggests that while all groups showed improvement, Group 2 had the greatest overall decrease in pain levels by the end of the study period. The use of dynamic MRI played a critical role in differentiating patellar alignment subtypes, which helped to identify biomechanical contributors to pain and treatment response. For example, the more substantial pain reduction observed in Group 2 could be attributed to the greater initial malalignment and subsequent biomechanical adaptations targeted by the intervention. Dynamic MRI therefore has potential as a diagnostic tool to personalize treatment, particularly in patients with complex patellofemoral biomechanics.

Traditionally, lateral patellar tracking in individuals with patellofemoral pain (PFP) was linked to a weakness or underdevelopment of the vastus medialis (VM) muscle compared to the larger and stronger vastus lateralis (VL) muscle [90,91,92] with an increased medial patello–femoral distance [14]. Even though there are papers supporting this theory, there are others that do not show any differences in thickness or electromyographic activation [75,76,93]. To understand why there is a discrepancy between research on the morpho-functional characterization of the vastus obliquus medialis and vastus lateralis and the numerous studies supporting the strengthening of the vastus medialis oblique in PFP, future studies should focus on analyzing proprioception, which changes in this pathology, to verify any potential differences during movements involving greater or lesser engagement of the vastus medialis or vastus lateralis. Following the traditional therapy focused on the vastus medialis, in this study, LMV was applied via three probes directly on the muscle belly of the vastus medialis, as shown in Figure 1. LMV was shown to induce a reduction in muscle fatigue and an improvement in muscle contraction properties [94]. LMV can also induce mechanotransduction by generating targeted muscle vibrations. At a frequency of 300 Hz, these vibrations were shown to enhance muscle tone and trophism through proprioceptive stimulation of neuromuscular spindles, Pacinian corpuscles, Golgi tendon organs, and type III-IV muscle mechanoreceptors [95]. The application of LMV provides a significant reduction in pain as it is indicated for pain control due to its ability to specifically activate highly myelinated fibers [96,97,98]. Thus, with a strong homotopic gating effect [99] and review highlighted the promising effects of LMV in improving pain, stiffness, function, and knee range of motion (ROM) in individuals with knee osteoarthritis (OA) [40].

The findings regarding patellar alignment are also noteworthy. Patients with more severe malalignment (e.g., lateral subluxation or dislocation) experienced a greater reduction in pain compared to those with less severe alignment issues. A past paper suggested that McConnell taping modifies patellar alignment but does not improve proprioception and motor functions, unlike kinesiotaping [25]. The application of kinesiotaping to facilitate VMO activation may increase eccentric VMO activity in adults with anterior knee pain during stair descent [100]. Our results could suggest that the combination of LMV and KMcCM may be particularly effective not only in addressing biomechanical abnormalities associated with patellar maltracking but also in enhancing VMO muscle contraction, leading to more substantial improvements in these patients.

## 5. The Strengths and Weaknesses of This Study

Despite these promising results, several limitations must be acknowledged. The study’s observational design may introduce selection bias, as only patients who completed the full course of treatment were included in the analysis. Furthermore, this study lacks a control group, which limits the ability to isolate the natural history of PFPS or placebo effects from the observed improvements, and the absence of separate groups for LMV therapy and KMcCM prevents determining the individual contribution of each modality to symptom reduction. Future randomized controlled trials (RCTs) would be necessary to confirm these findings and establish a causal relationship. Although efforts were made to standardize the LMV intensity and KMcCM application, minor inter-session variability due to manual application cannot be fully excluded. Future studies should also focus on investigating different power levels or frequencies in the field of physical and rehabilitation medicine to determine which energy setting is the most effective [101,102,103,104]. Moreover, with the advancement of artificial intelligence and machine learning methods, future studies should explore this aspect, especially in physical and rehabilitation medicine and tendinopathies, similarly to previously developed studies [105,106,107,108].

Overall, this study contributes to the growing body of evidence supporting the use of multimodal therapeutic approaches for PFPS, particularly LMV as a cost-effective alternative to whole-body vibration therapy. It also contributes to research on combined therapies to maximize the positive effects of various treatments. In recent years, these approaches were developed thanks to the wide availability of physical modalities in physical and rehabilitation medicine [109,110,111,112,113,114,115]. Future studies should focus on longer-term outcomes, the potential for relapse, and the exploration of other adjunctive therapies to enhance treatment effectiveness, especially considering the gender and age-related differences observed in this study.

## 6. Conclusions

The combination of local muscle vibration (LMV) and kinesiotaping applied using the McConnell method (KMcCM) was effective in reducing pain and improving lower limb function in patients with patellofemoral pain syndrome (PFPS). Significant improvements were observed in both VAS and KOOS, indicating benefits in pain reduction and functional recovery. Younger patients demonstrated greater improvements, particularly females under 60 compared to older women, in response to the combined treatment, suggesting that age and gender influence recovery. Age-related physiological changes, such as hormonal differences, may affect recovery, with estrogen enhancing pain modulation and recovery. Gender differences in pain perception and treatment response are also shaped by biological, psychological, and social factors. Further research is needed to explore these mechanisms and assess long-term effects across diverse populations.

## Figures and Tables

**Table 1:** Comparison of baseline variables and outcomes measures before and after treatment. Values reported as mean ± standard deviation for continuous variable and as distribution for categoric variable and *p* value.

Variable	Value
Age (years)	66 ± 12.2
VAS T0	6 ± 1.4
VAS T1	2.8 ± 1.9
KOOS T0	45.6% ± 9.6%
KOOS T1	72.7% ± 15.3%
Gender (Male/Female)	14/38
Patellar Alignment (Normal)	15 (28.8%)
Patellar Alignment (Tilt/Hyperpressure)	24 (46.2%)
Patellar Alignment (Subluxation/Dislocation)	13 (25%)
Medial patellofemoral ligament thickness	2 ± 0.7
Cartilages Medial Thickness	1.8 ± 0.4
Cartilages Lateral Thickness	2 ± 0.5
Medial Meniscus Binay Value (healthy/injured)	35/17
Lateral Meniscus Binay Value (healthy/injured)	41/11
Age Group 1 < 60	13
Age Group 2 ≥ 60	39
Delta KOOS Group 1 < 59%	11
Delta KOOS Group 2 ≥ 60%	41

VAS: Visual Analog Scale; KOOS: Knee Injury and Osteoarthritis Outcome Score.

**Table 2:** GLM results for VAS T0-T1 and age group interaction effect.

Age Group	VAS T0 (Mean ± SD)	VAS T1 (Mean ± SD)	*p*-Value (Time)	Interaction Effect (F, *p*, η^2^)
<60 years	5.6 ± 1.3	1.8 ± 1.3	<0.001	F(1, 50) = 3.71, *p* < 0.05, η^2^ = 0.07
≥60 years	6 ± 1.4	3.2 ± 2	<0.01	

**Table 3:** GLM results for VAS T0-T1 and delta KOOS interaction effect.

Delta KOOS Group	VAS T0 (Mean ± SD)	VAS T1 (Mean ± SD)	*p*-Value (Time)	Interaction Effect (F, *p*, η^2^)
Group 1 < 59%	6.6 ± 1.1	2.7 ± 1.2	<0.001	F (1, 50) = 4.3, *p* < 0.05, η^2^ = 0.08
Group 2 ≥ 60%	5.7 ± 1.4	2.9 ± 2	<0.01	

**Table 4:** GLM results for VAS T0-T1 and Patellar Dynamic Alignment interaction effect.

Patellar Alignment Group	VAS T0 (Mean ± SD)	VAS T1 (Mean ± SD)	*p*-Value (Time)	Interaction Effect (F, *p*, η^2^)	Post Hoc
Neutral (Group 0)	5.3 ± 1.1	3.1 ± 2	0.01	F(2, 49) = 3.83, *p* = 0.028, η^2^ = 0.135	Group 2 vs. Group 0 (*p* < 0.05)
Hyperpressure (Group 1)	6.1 ± 1.4	2.8 ± 2	<0.001		Group 2 vs. Group 1 (*p* < 0.05)
Subluxation/Dislocation (Group 2)	6.2 ± 1.5	2.5 ± 1.6	0.02		

**Table 5:** GLM results on female subgroup for VAS T0-T1 and age group interaction effect.

Age Group	VAS T0 (Mean ± SD)	VAS T1 (Mean ± SD)	*p*-Value (Time)	Interaction Effect (F, *p*, η^2^)
<60 years	5.7 ± 1.6	1.3 ± 1.2	<0.001	F(1, 36) = 4.9, *p* < 0.03, η^2^ = 0.12
≥60 years	6.2 ± 1.5	3.2 ± 2.1	<0.01	

## Data Availability

The datasets used and the data analyzed during the current study will be made available upon reasonable request to the corresponding author (G.S. (Gabriele Santilli)).
